# Supplementary material for: Phenformin Down-Regulates c-Myc Expression to Suppress the Expression of Pro-Inflammatory Cytokines in Keratinocytes
Source: Cells. 2022 Aug 5;11(15):2429. doi: 10.3390/cells11152429 (PMC9368166; doi:10.3390/cells11152429)
Supplement: Supplementary file 1 [file cells-11-02429-s001.zip › cells-1794648-supplementary.pdf]

Supplementary Figures and Tables

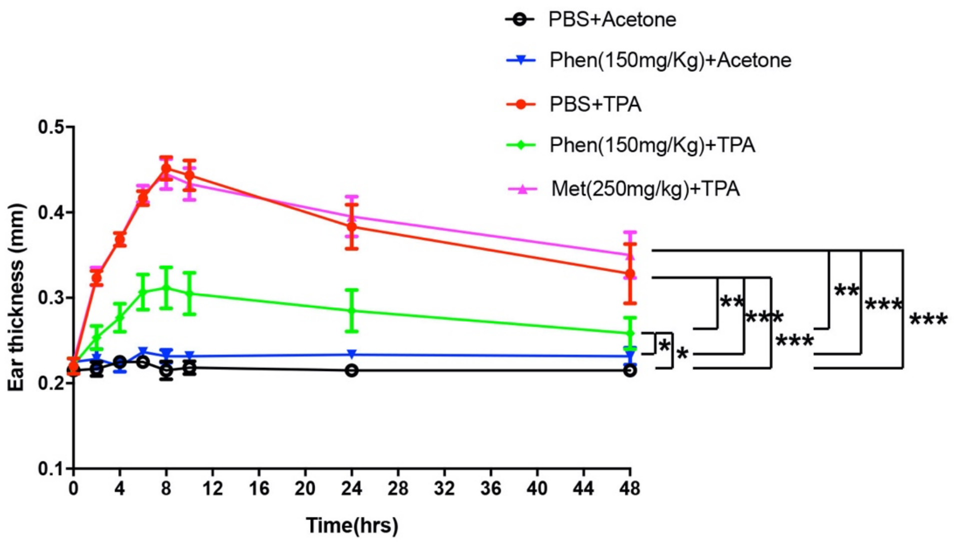

Supplementary Figure S1. Phenformin but not metformin suppresses the skin acute inflammatory response induced by TPA.

Mice were randomly divided into three groups, treated with PBS, phenformin (150 mg/Kg) or metformin (250 mg/Kg) followed by the topical application of Acetone on the left ears or TPA on the right ears, after which the ear thickness was measured at different time points as indicated. Two-way ANOVA with correction for multiple pairwise comparisons was used for statistical analysis of differences between two groups as indicated., n = 3, Standard error bars are provided, $*p<0.05$; $**p<0.01$; $***p<0.005$.

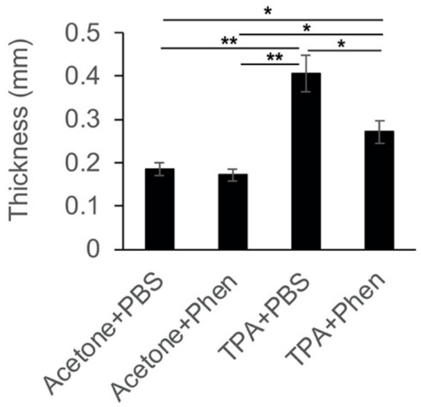

Supplementary Figure S2. Phenformin reduces the ear thickness increased by TPA treatment.

Quantification of ear thickness for HE stains in Fig. 1C. Student's t test analysis was used for all quantification data to compare two groups as indicated, n = 3, Standard error bars are provided, *p<0.05; **p<0.01.

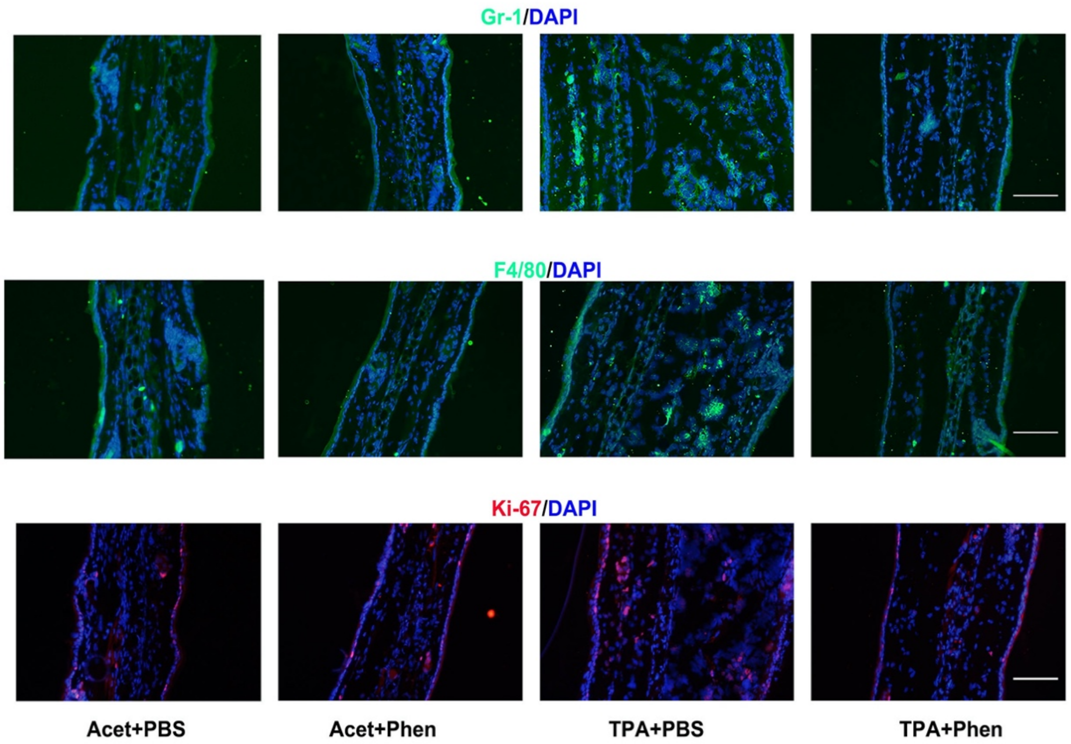

Supplementary Figure S3. Phenformin reduces the infiltration of immune cells induced by TPA treatment in the dermis.

Sections derived from mouse ear skin were treated with different conditions: Acetone plus PBS (Acet+PBS), Acetone plus phenformin (Acet+Phen), TPA plus PBS (TPA+PBS) and TPA plus phenformin (TPA+Phen) were analyzed for IF staining of Gr-1 (upper panels, green), F4/80 (middle panels, green) and Ki67 (bottom panels, red); DAPI is used as a counterstain for nuclei. Higher magnification images are shown in **Fig. 1 D,F,H**.

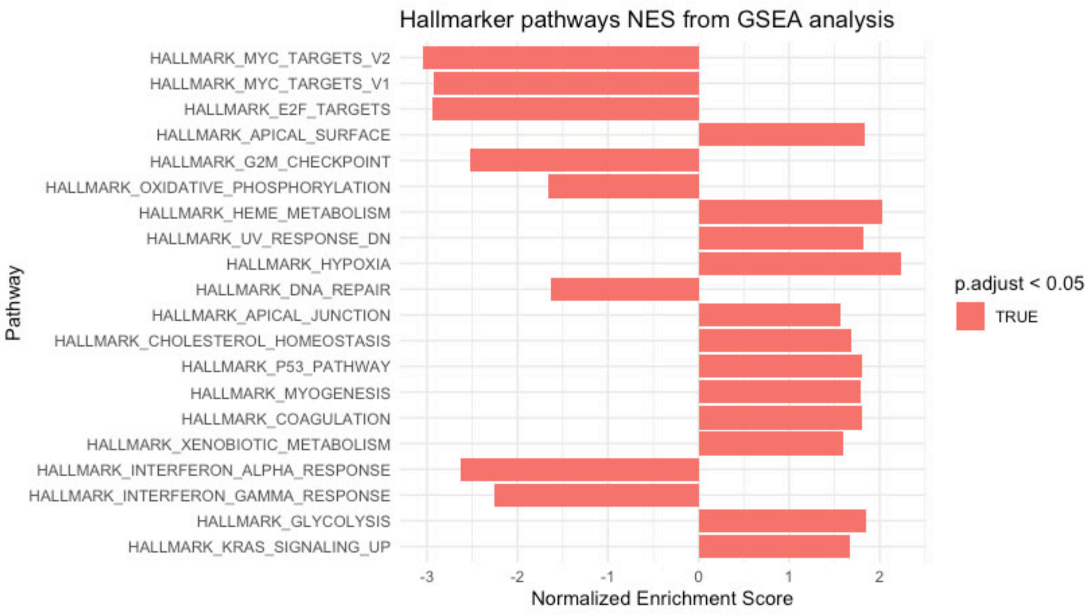

Supplementary Figure S4. List of the Top 20 enriched Hallmark pathways from GSEA analysis.

Gene Set Enrichment Analysis (GSEA) of DEGs identified the Top 20 enriched Hallmark pathways including 12 upregulated (normalized enrichment score>0) and 8 downregulated pathways (normalized enrichment score<0) according to the normalized enrichment score ($p < 0.05$).

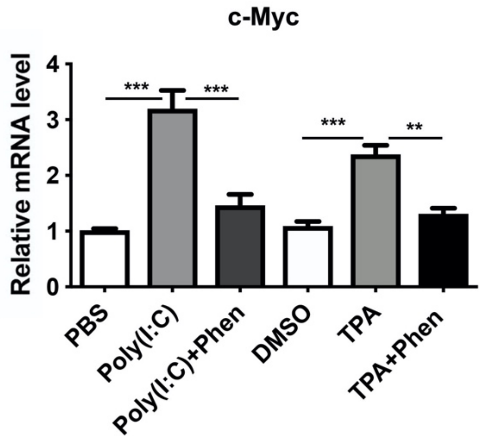

Supplementary Figure S5. Phenformin suppresses c-Myc expression induced by either Poly (I:C) or TPA in keratinocytes.

Total mRNAs were extracted from keratinocytes treated with 10 μg/ml Poly(I:C) or with 5 μg/ml TPA combined with 1 mM phenformin together with the corresponding control (PBS or DSMO), and qRT-PCR analysis of c-Myc expression, which was normalized to levels of 36B4 mRNA. Student's t test analysis was used to compare each different concentration group as indicated, n = 3, standard deviation bars are shown, $^{**}p<0.01$; $^{***}p<0.005$.

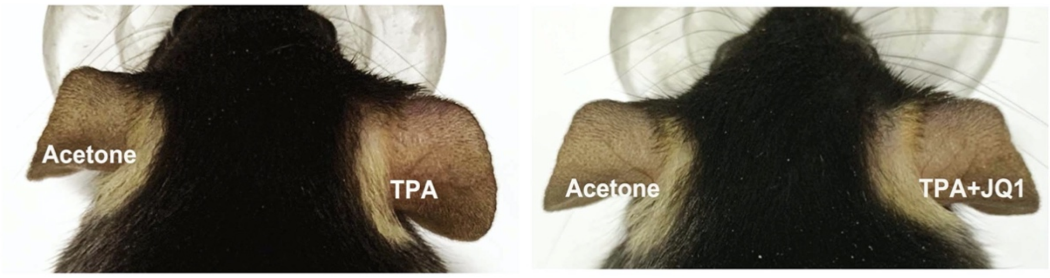

Supplementary Figure S6. The c-Myc inhibitor JQ1 suppresses the skin acute inflammatory response induced by TPA treatment.

Representative images of mouse ears at 8 h after treatment with the following combinations: Left ears: Acetone (Negative control); Right ears: TPA (positive control) or TPA+JQ1 (TPA+JQ1).

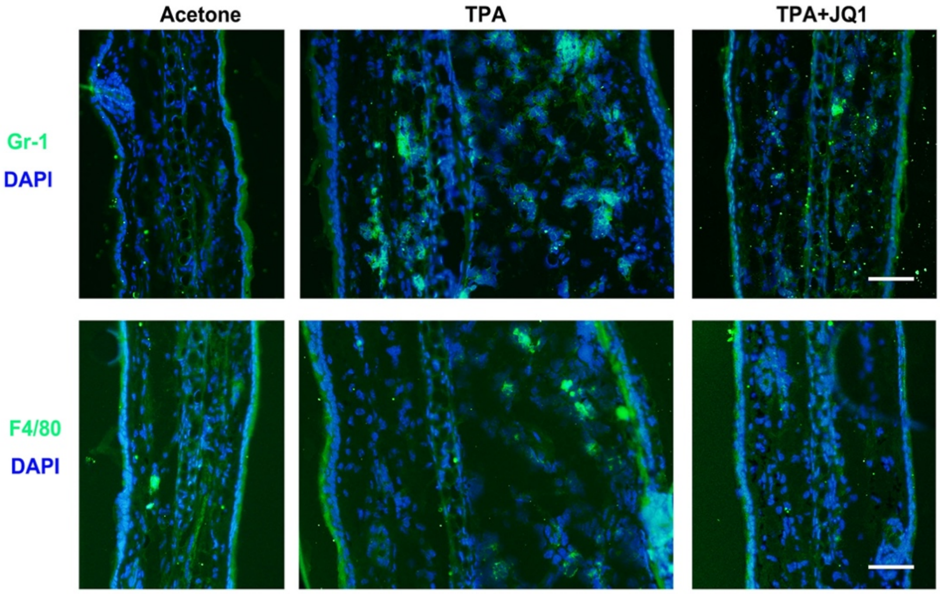

Supplementary Figure S7. The c-Myc inhibitor JQ1 decreases the infiltration of immune cells induced by TPA treatment in the dermis.

Sections derived from mouse ear skin treated with different conditions: Acetone, TPA and TPA+ JQ1 (details are described in the Figure 5 legend) were analyzed for IF staining of Gr-1 (upper panels, green) and F4/80 (lower panels, green). DAPI is used as a counterstain for nuclei. Higher magnification images are shown in **Figure. 5I**.

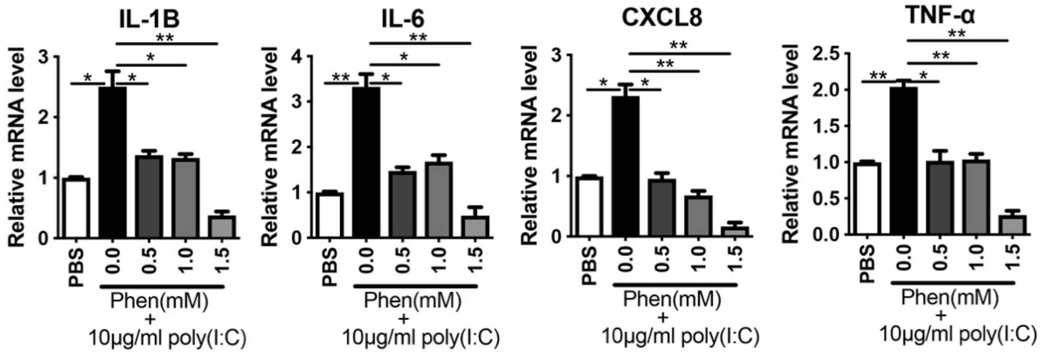

Supplementary Figure S8. Phenformin suppresses pro-inflammatory cytokine expression induced by Poly (I:C) in dermal fibroblasts.
Primary dermal fibroblasts were isolated from human foreskin tissues, and were cultured in 10% FCS in DMEM. Passage 3 dermal fibroblasts at 80% confluence were treated with 10 μg/ml Poly(I:C) together with different concentrations (0.0 mM, 0.5 mM, 1 mM, 1.5 mM) of phenformin or PBS alone as a negative control for 24 h, after which the cells were collected for qRT-PCR analysis of IL-1β(IL-1B), IL-6, IL-8(CXCL8) and TNF-α expression levels, which were normalized to levels of 36B4 mRNA. Student's t test analysis was used to compare two different groups as indicated, n = 3, standard deviation bars are shown, $*p<0.05$, $**p<0.01$.

Supplementary Table S1： Oligos sequences used for RT-PCR analysis.

Gene	Human
h36B4-1	Forward：5’- GCAATGTTGCCAGTGTCTGT -3’<br>Reverse：5’- GCCTTGACCTTTTCAGCAAG -3’
IL-6	Forward：5’- TTCTTGGGACTGATGCTGGT -3’<br>Reverse：5’- CCTCCGACTTGTGAAGTGGT -3’
CXCL8	Forward：5’- CTTTCCAATTCGGGAGACCT -3’<br>Reverse：5’- GGCATCACTGCCTGTCAAG -3’
IL-1B	Forward：5’- CAACAAGTGGTGTTCTCCATGTC -3’<br>Reverse：5’- ACACGCAGGACAGGTACAGA -3’
TNF-α	Forward：5’- CCACCACGCTCTTCTGTCTA -3’<br>Reverse：5’- TTGTGAGTGTGAGGGTCTGG -3’
IL-12B	Forward：5’- GGTATCACCTGGACCTTGGA -3’<br>Reverse：5’- GCTTAGAACCTCGCCTCCTT -3’
IL-23B	Forward：5’- CCGCTTCAAAATCCTTCGCA -3’

	Reverse：5'- TCTGAGTGCCATCCTTGAGC -3'
CCL2/MCP1	Forward：5'- CTCGCCTCCAGCATGAAAGT -3'<br>Reverse：5'- GGTGACTGGGGCATTGATTG -3'
CXCL16	Forward：5'- CACGAGGTTCCAGCTCCTTT -3'<br>Reverse：5'- CCACAATCCCCGAGTAAGCA -3'
TGF-B1	Forward：5'- CGACTCGCCAGAGTGGTTAT -3'<br>Reverse：5'- TGCAGTGTGTTATCCCTGCT -3'
AMPKα1	Forward：5'- GCACCTTCGGCAAAGTGAAG -3'<br>Reverse：5'- CCTACCACATCAAGGCTCCG -3'
AMPKα2	Forward：5'- CAGTGTGTTCTTATTCAACATCTCA -3'<br>Reverse：5'- ACTGCCACTTTATGGCCTGT -3'
C-Myc	Forward：5'- CTCCTACGTTGCGGTCACAC -3'<br>Reverse：5'- TGATGAAGGTCTCGTCGTCC -3'
CDK4	Forward：5'- TTGCATCGTTCACCGAGATC -3'<br>Reverse：5'-CTGGTAGCTGTAGATTCTGGCCA -3'
DDX18	Forward：5'- ATGTCACACCTGCCGATGAAA -3'<br>Reverse：5'- CCCTGAAACTTTAGGTTCCGC -3'
APEX1	Forward：5'-GCCCACTCAAAGTTTCTTAC -3'<br>Reverse：5'-TGTGCCACATTGAGGTCTCC -3'
CAD	Forward：5'-CTCACTGATCCCTCCTACAA -3'<br>Reverse：5'-GTGGATACGACACTGGGATA -3'
NOP16	Forward：5'-GCGTCTGAACCGGAATGCTC -3'<br>Reverse：5'-CCAGGTTCTGCCGTACCGAT -3'

Supplementary Table S2. siRNA sequences used in this study

Myc (c-Myc)	Myc-homo-1344	Sense	5'-GAG GAU AUC UGG AAG AAA UTT-3'
		Antisense	5'-AUU UCU UCC AGA UAU CCU CTT-3'
	Myc-homo-1729	Sense	5'-GCU UGU ACC UGC AGG AUC UTT-3'
		Antisense	5'-AGA UCC UGC AGG UAC AAG CTT-3'
	Myc-homo-1982	Sense	5'-GGA AGA AAU CGA UGU UGU UTT-3'
		Antisense	5'-AAC AAC AUC GAU UUC UUC CTT-3'
